# Serosurvey of Blood Donors to Assess West Nile Virus Exposure, South-Central Spain

**DOI:** 10.3201/eid3007.240450

**Published:** 2024-07

**Authors:** Mario Frías, Javier Caballero-Gómez, Ana Vázquez, Elena Madrigal, Francisco Ruiz-Fons, Marina Gallo, Laura Herrero, María Jarilla, Ignacio García-Bocanegra, Antonio Rivero-Juárez Antonio Rivero

**Affiliations:** CIBERINFEC, Madrid, Spain (M. Frías, J. Caballero-Gómez, M. Gallo, L. Herrero, I. García-Bocanegra, A. Rivero-Juárez, A. Rivero);; Universidad de Córdoba, Córdoba, Spain (M. Frías, J. Caballero-Gómez, M. Gallo, I. García-Bocanegra, A. Rivero-Juárez, A. Rivero);; CIBERESP, Madrid (A. Vázquez);; Instituto de Salud Carlos III, Madrid (A. Vázquez, L. Herrero);; Hospital General Universitario de Ciudad Real, Ciudad Real, Spain (E. Madrigal);; Instituto de Investigación en Recursos Cinegéticos, Ciudad Real (F. Ruiz-Fons);; Hospital Universitario Reina Sofía, Córdoba (M. Jarilla)

**Keywords:** West Nile virus, virus neutralization test, viruses, vector-borne infections, zoonoses, serosurvey, blood donors, Spain, Ciudad Real

## Abstract

We analyzed West Nile Virus (WNV) exposure from 1,222 blood donors during 2017–2018 from an area of south-central Spain. Results revealed WNV seroprevalence of 0.08% (95% CI 0.004%–0.4%) in this population. Our findings underscore the need for continued surveillance and research to manage WNV infection in this region.

West Nile virus (WNV), a member of the family Flaviviridae, genus *Orthoflavivirus*, is classified within the Japanese encephalitis virus (JEV) serocomplex (*1*). It is the most widespread arbovirus globally, primarily because of the abundance and broad distribution of its main competent vector, mosquitoes belonging to the genus *Culex* (*2*). During the past 2 decades, WNV has led to epidemic outbreaks with a substantial proportion of severe cases in Europe, emerging as a considerable threat to public and animal health in these regions. Nonetheless, very limited information exists on seroprevalence in the general population, hindering a comprehensive understanding of the virus’ epidemiologic landscape.

In Spain, WNV is considered endemic because of conducive conditions for virus maintenance and circulation, including diverse bird reservoirs, geographic characteristics such as migratory bird routes, and specific climatic conditions. Since a notable outbreak reported in 2020, the virus has produced human cases annually (*3*), demonstrating the spread of the virus in the country (*4*). Therefore, vigilant surveillance in new risk areas is imperative to anticipate potential human health emergencies. Studies in vectors and animal hosts in south-central Spain have underscored the region’s potential as a hotspot zone (*5*–*7*). Within this area, the province of Ciudad Real, where no human WNV cases have been reported to date, serves as an ideal scenario for assessing circulation of the virus in the general population. We conducted a serosurvey in blood donors to investigate WNV exposure in the general population of this region in Spain, shedding light on the transmission dynamics of this emergent virus.

We conducted a retrospective cross-sectional study to analyze the seroprevalence of WNV in serum samples collected from blood donors at the Transfusion Center of the Hospital General Universitario de Ciudad Real (south-central Spain) (Figure) during 2017–2018 (Appendix). We selected and analyzed blood from 1,222 donors (Appendix Table 1). Sex and age data were not available for 129 (10.5%) donors. Of the 1,093 donors for whom information was available, 571 (52.2%) were men and 522 (47.8%) women. The age of the donors was categorized into 3 classes: <30 years (21.8% of samples), 30–50 years (34.8%), and >50 years (32.7%). Nineteen (1.6%) of the samples reacted positively to the IgG WNV ELISA. We administered an epidemiologic survey to the 19 ELISA-positive donors; 16 donors responded (Appendix Table 2). 

**Figure:**
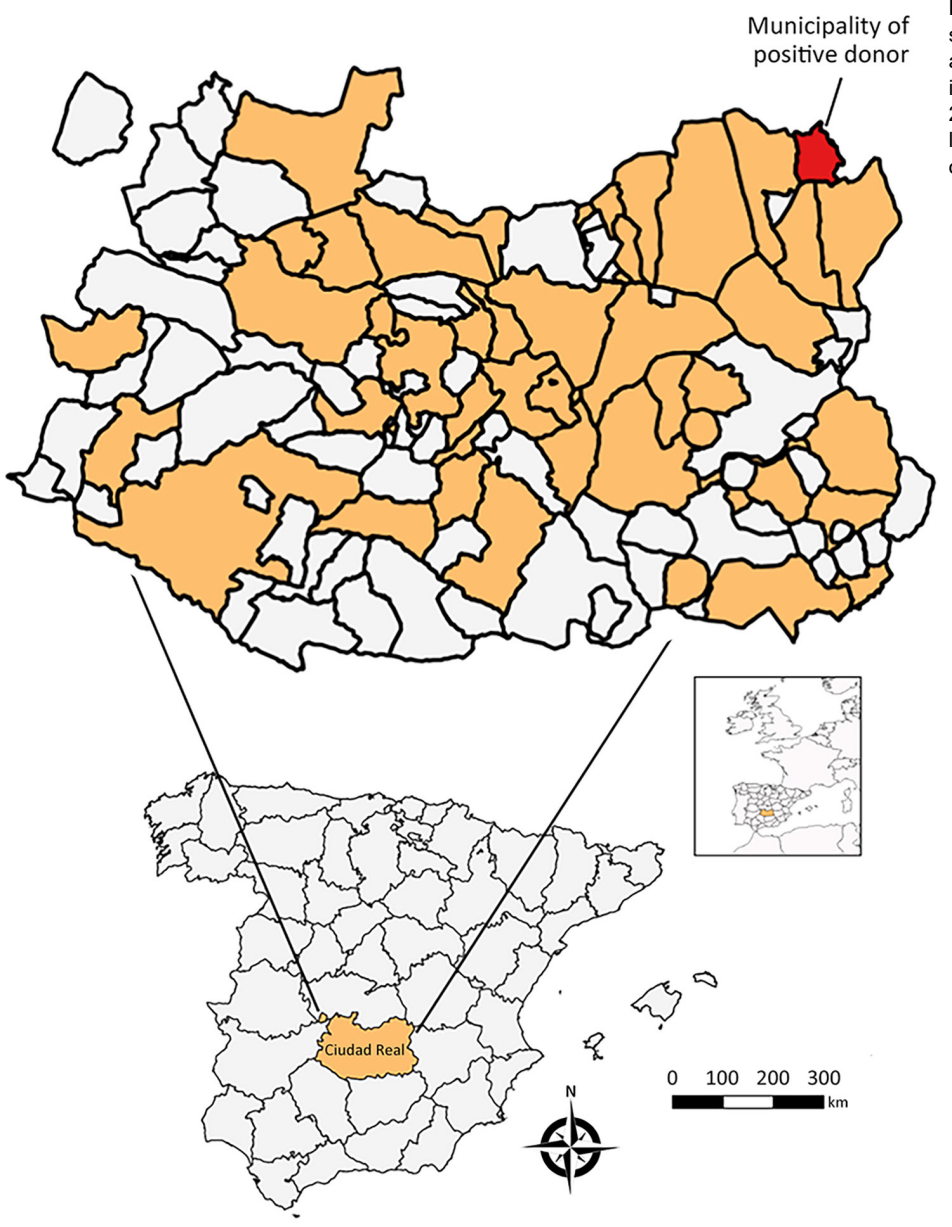
Locations sampled in serosurvey of blood donors to assess West Nile virus exposure in the general population, Spain, 2017–2018. Inset maps show location of study area in Spain and of Spain in Europe.

We analyzed all ELISA-positive samples by using a virus neutralization test (VNT) (Appendix Table 2). Regarding WNV, ELISA reactivity was only confirmed by VNT in 1 donor who showed a titer of 1/256, which indicated a seroprevalence of 0.08% (95% CI 0.04%–0.4%) for WNV. This donor declared that he had not traveled outside of Spain and therefore did not receive any vaccine against yellow fever virus, tick-borne encephalitis virus, or Japanese encephalitis virus.

In Europe, no seroepidemiologic studies have been conducted since 2013; therefore, our study would provide valuable insights into the current status of WNV exposure. Our study encompasses a vast region of south-central Spain and marks initial identification of seropositivity in humans in this specific region of Spain, indicating a broad spread of the virus. In Spain, recent serosurveys are lacking; 2 studies were conducted in Catalonia in 2001 (0.2%) (*8*) and 2011 (0.12%) (*9*), and another was conducted in the province of Sevilla in 2006 (0.6%) (*10*). In the past 3 years, the regions of those studies have experienced large WNV outbreaks, similar to that which occurred in summer of 2020 (*3*) or the first description of clinical cases in Catalonia in 2022 and 2023 (*4*). This development suggests greater exposure to the virus than in the previous decade and highlights the need to carry out new serosurveys in the general population that enable collection of updated data.

The observed seroprevalence among blood donors from south-central Spain in our study suggests a low exposure (0.08%) to WNV in the general population within this spatiotemporal context. Of note, the number of WNV cases in Spain has been on the rise in recent years, being detected even in areas where previously no evidence of WNV circulation existed, suggesting that WNV has been expanding during recent years and that outbreaks can be expected in areas not currently considered endemic for WNV.

In our study, and in line with other studies (*9*), a high percentage (94.7%) of ELISA-positive WNV samples could not be confirmed as positive for specific antibodies. This finding highlights the need to perform additional neutralization tests against other flaviviruses in the serosurvey studies. The absence of an ELISA test with high sensitivity and, more crucially, specificity for WNV, limits the design of large-scale population serosurvey studies. Urgent efforts are required to address this limitation.

In conclusion, our study indicated seropositivity in the south-central region of Spain. In this way, reporting cases in Spain may be plausible even in areas not at high risk, highlighting the importance of ongoing surveillance and research to manage WNV infection in this region.

Appendix Additional information about serosurvey of blood donors to assess West Nile virus exposure, South-Central Spain.
