# Supplementary material for: Serosurvey of Blood Donors to Assess West Nile Virus Exposure, South-Central Spain
Source: Emerg Infect Dis. 2024 Jul;30(7):1496–8. doi: 10.3201/eid3007.240450 (PMC11210648; doi:10.3201/eid3007.240450)
Supplement: Appendix — Additional information about serosurvey of blood donors to assess West Nile virus exposure, South-Central Spain. [file 24-0450-Techapp-s1.pdf]

Article DOI: https://doi.org/10.3201/eid3007.240450

EID cannot ensure accessibility for supplementary materials supplied by authors. Readers who have difficulty accessing supplementary content should contact the authors for assistance.

Serosurvey of Blood Donors to Assess West Nile Virus Exposure, South-Central Spain

Appendix

Selection of Blood Donors and Sample Size Calculation

The blood donor samples comprised 10,232 serum samples, providing extensive coverage across the Ciudad Real province (42 municipalities) (Figure, https://wwwnc.cdc.gov/EID/article/30/7/24-0450-F1.htm). In a cross-sectional epidemiologic approach, we sought to determine the overall exposure rate of the human population in the study area by employing a representative subset. To achieve this, we calculated the minimum sample size necessary to estimate the prevalence of WNV exposure at the finest spatial resolution available for blood donor information, namely the municipality. Appendix Table 1 shows the 2020 human census and sample size per municipality. The required sample size for estimating the prevalence of exposure at the municipal level, considering an expected seroprevalence of 1.7% (based on findings a in Greek seroprevalence study [*1*]), was determined to be 26 donors per municipality, with a 5% of precision and 95% confidence interval. Sample selection was conducted in a balanced manner, according to the age (three classes: <30 years old, 30–50 years old, and >50 years old) and sex of donors per municipality.

Serologic Methods

To detect IgG antibodies against WNV, the commercial ELISA West Nile Virus IgG DxSelect (Focus Diagnostics Inc., Cypress, California) were used. ELISA-positive samples were subjected to additional testing using a virus neutralization test (VNT) for WNV lineage 1 (WNV-1), Usutu (USUV) and Tick-borne encephalitis (TBEV). VNT is described in detail elsewhere

(*2*). We selected these viruses because are endemic in Europe, in fact, in recent years, the areas in Europe reporting flavivirus infections and specifically WNV, USUV, or TBEV have significantly increased (*3*). WNV and USUV are endemic in Spain and although no autochthonous TBEV human cases have been detected, recent animal seroprevalence studies indicate the possibility of TBEV circulation in horses and dogs from several areas in Spain (*4*,*5*). The titers of neutralizing antibodies were defined as the highest serum dilution that showed >90% neutralization of the virus challenge. Neutralizing antibody titers ≤1:8 were considered negative.

VNT used Vero E6 cells and the virus strains HU6365/08 (WNV-1, GenBank: JF707789.1), Neudorfl (TBE) and HU10279/09 (USUV, GenBank accession no. KX268472.1). A sample was considered WNV seropositive when it was reactive via ELISA in the VNT assay the antibody titer obtained against WNV was 4 times higher than the antibody titer obtained against another of the three tested flavivirus.

Epidemiologic Survey

ELISA-positive donors underwent a telephone interview conducted by the blood bank staff, which an epidemiologic survey was performed to obtain information about their country of birth, WNV-endemic countries visited, vaccination history, and specific vaccinations against other flaviviruses, such as yellow fever virus (YFV) and Japanese encephalitis virus (JEV), due to the potential for cross-reaction in the ELISA results. Only the WNV-seropositive donor confirmed by VNT was informed of the results.

Ethics Considerations

This study was designed and performed according to the Helsinki Declaration. All samples were integrated into 'Biobanco del Sistema Sanitario Pública de Andalucía (Nodo Hospital Universitario Reina Sofía-IMIBIC)'. All donors signed an informed consent form. The CEIC (Clinical Research Ethics Committee) of Hospital de Ciudad Real approved the collection of samples.

Supplementary Results

Two donors with positive results in the IgG WNV ELISA assay and not confirmed by VNT against any of the three tested flaviviruses, were natives from countries where the virus is endemic (Colombia and Ecuador) and frequently traveled to their countries of origin. Therefore, the positive results from the WNV IgG ELISA are due to a previous flavivirus infection endemic to this area, such as dengue and/or Zika viruses. None of the ELISA-positive donors showed neutralizing antibodies against USUV or TBEV.

References

1. Hadjichristodoulou C, Pournaras S, Mavrouli M, Marka A, Tserkezou P, Baka A, et al.; MALWEST Project. West Nile virus seroprevalence in the Greek population in 2013: a nationwide cross-sectional survey. PLoS One. 2015;10:e0143803. PubMed https://doi.org/10.1371/journal.pone.0143803

2. Macias A, Martín P, Pérez-Olmeda M, Fernández-Martínez B, Gómez-Barroso D, Fernández E, et al. West Nile virus emergence in humans in Extremadura, Spain 2020. Front Cell Infect Microbiol. 2023;13:1155867. PubMed https://doi.org/10.3389/fcimb.2023.1155867

3. Barzon L. Ongoing and emerging arbovirus threats in Europe. J Clin Virol. 2018;107:38–47. PubMed https://doi.org/10.1016/j.jcv.2018.08.007

4. García-Bocanegra I, Jurado-Tarifa E, Cano-Terriza D, Martínez R, Pérez-Marín JE, Lecollinet S. Exposure to West Nile virus and tick-borne encephalitis virus in dogs in Spain. Transbound Emerg Dis. 2018;65:765–72. PubMed https://doi.org/10.1111/tbed.12801

5. Vanhomwegen J, Beck C, Desprès P, Figuerola A, García R, Lecollinet S, et al. Circulation of zoonotic arboviruses in equine populations of Mallorca Island (Spain). Vector Borne Zoonotic Dis. 2017;17:340–6. PubMed https://doi.org/10.1089/vbz.2016.2042

Appendix Table 1. Demographic characteristics

Municipality	Census persons (year 2020)	No. donations available	No. donors included
Agudo	1,657	72	41
Alcázar San Juan	30,766	951	28
Aldea del Rey	1,631	30	29
Almadén	5,200	91	33
Almagro	8,905	448	31
Almodóvar	5,983	63	31
Argamasilla de Alba	6,955	216	31
Bolaños de Calatrava	12,019	273	27
Calzada de Calatrava	3,630	141	31
Campo de Criptana	13,312	122	30
Carrión de Calatrava	3,099	21	21
Castellar de Santiago	1,862	78	31
Ciudad Real (Capital province)	75,504	643	30
Corral de Calatrava	1,117	35	31
Daimiel	17,916	687	31
Fuente El Fresno	3,208	137	31
Herencia	8,456	230	31
Malagón	7,881	209	29
Manzanares	17,962	599	31
Membrilla	5,942	161	30
Miguelturra	15,498	189	28
Montiel	1,294	20	19
Moral de Calatrava	5,208	163	31
Pedro Muñoz	7,285	137	31
Picón	668	26	26
Piedrabuena	4,379	183	31
Pozuelo de Calatrava	3,586	105	28
Puerto Lápice	891	30	30
Puertollano	46,607	1154	26
Retuerta del Bullaque	927	22	22
Robledo	1,053	20	20
San Carlos del Valle	1,109	63	31
Santa Cruz de Mudela	4,085	92	26
La Solana	15,419	226	31
Tomelloso	36,168	1,019	30
Torralba de Calatrava	2,966	48	30
Valdepeñas	30,252	829	28
Villahermosa	1,790	39	31
Villamanrique	1,128	22	22
Villanueva de los infantes	4,869	128	31
Villarrubia de los ojos	9,762	459	31
Villarta de San Juan	2,739	51	31
Total	430,688	10,232	1,222

Appendix Table 2. Serology and epidemiology of ELISA-positive donors

ID	Donation date	Age (years)	Sex	Country of origin	Travel in WNV-endemic area	Vaccines	IgG ELISA	VNT WNV1	VNT USUV	VNT TBEV
WNV01	Feb-18	30–50	Male	Spain	No	No	Positive	Negative	Negative	Negative
WNV02	Feb-18	30–50	Female	Ecuador	Ecuador	NA	Positive	Negative	8-Jan	Negative
WNV03	Feb-18	30–50	Male	Spain	No	No	Positive	Negative	Negative	Negative
WNV04	Feb-18	30–50	Male	Colombia	Colombia	No	Positive	Negative	Negative	Negative
WNV05	Feb-18	>50	Male	Spain	No	No	Positive	Negative	Negative	Negative
WNV06	Jan-18	30–50	Female	Spain	No	No	Positive	Negative	Negative	Negative
WNV07	Jan-18	<30	Male	Spain	No	No	Positive	1/256	1/8_1/16	Negative
WNV08	Jan-18	<30	Male	Spain	NA	NA	Positive	Negative	Negative	Negative
WNV09	Jan-18	>50	Female	Spain	No	No	Positive	Negative	Negative	Negative
WNV10	Jan-18	<30	Male	Spain	No	No	Positive	Negative	Negative	Negative
WNV11	Feb-18	<30	Male	Spain	NA	NA	Positive	Negative	Negative	Negative
WNV12	Jan-18	<30	Male	Spain	No	No	Positive	Negative	Negative	Negative
WNV13	Feb-18	>50	Male	Spain	No	No	Positive	Negative	Negative	Negative
WNV14	Feb-18	>50	Female	Spain	No	No	Positive	Negative	Negative	Negative
WNV15	Feb-18	<30	Female	Spain	No	No	Positive	Negative	Negative	Negative
WNV16	Feb-18	<30	Male	Spain	No	No	Positive	Negative	Negative	Negative
WNV17	Apr-18	>50	Male	Spain	No	No	Positive	Negative	Negative	Negative
WNV18	Apr-18	<30	Female	Spain	No	No	Positive	Negative	Negative	Negative
WNV19	Apr-18	30–50	Female	Romania	Romania	No	Positive	Negative	Negative	Negative

NA, unavailable information.
